# Diagnostic value of the lumbar extension-loading test in patients with lumbar spinal stenosis: a cross-sectional study

**DOI:** 10.1186/1471-2474-15-259

**Published:** 2014-07-31

**Authors:** Naoto Takahashi, Shin-ichi Kikuchi, Shoji Yabuki, Koji Otani, Shin-ichi Konno

**Affiliations:** 1 Department of Orthopaedic Surgery, Fukushima Medical University School of Medicine, 1 Hikarigaoka, Fukushima 960-1295, Japan

**Keywords:** Lumbar extension-loading test, Gait-loading test, Lumbar spinal stenosis, Diagnostic value, Prospective cohort study, Neurogenic intermittent claudication, Subjective symptoms, Objective neurological findings, Provocation test, Pathology

## Abstract

**Background:**

The gait-loading test is a well known, important test with which to assess the involved spinal level in patients with lumbar spinal stenosis. The lumbar extension-loading test also functions as a diagnostic loading test in patients with lumbar spinal stenosis; however, its efficacy remains uncertain. The purpose of this study was to compare the diagnostic value of the lumbar extension-loading test with that of the gait-loading test in patients with lumbar spinal stenosis.

**Methods:**

A total of 116 consecutive patients (62 men and 54 women) diagnosed with lumbar spinal stenosis were included in this cross-sectional study of the lumbar extension-loading test. Subjective symptoms and objective neurological findings (motor, sensory, and reflex) were examined before and after the lumbar extension-loading and gait-loading tests. The efficacy of the lumbar extension-loading test for establishment of a correct diagnosis of the involved spinal level was assessed and compared with that of the gait-loading test.

**Results:**

There were no significant differences between the lumbar extension-loading test and the gait-loading test in terms of subjective symptoms, objective neurological findings, or changes in the involved spinal level before and after each loading test.

**Conclusions:**

The lumbar extension-loading test is useful for assessment of lumbar spinal stenosis pathology and is capable of accurately determining the involved spinal level.

## Background

Neurogenic intermittent claudication (NIC) is the most characteristic symptom in patients with lumbar spinal stenosis (LSS). Verbiest defined the pathomorphologic changes that take place in patients with LSS, specifically the encroachment of the canal by hypertrophied articular processes [1,2]. Subjective symptoms of LSS do not necessarily correspond with the spinal level of anatomical neural compression identified on magnetic resonance imaging (MRI) and/or myelography because of asymptomatic neural compression [3-5]. In addition, subjective symptoms and/or objective neurological findings (motor, sensory, and reflex) at rest are not consistent with the involved spinal level in patients with LSS. Therefore, it is difficult to accurately identify the involved spinal level based only on subjective symptoms and objective neurological findings at rest when multiple levels of stenosis are present on imaging. Because of the nature of NIC, a dynamic study involving gait-loading may be useful in the diagnosis of the spinal level truly responsible for producing symptoms in patients with LSS.

Confirmation of the involved spinal level and decompression of only the spinal level responsible for the symptoms are basic principles of successful LSS surgery. Less invasive surgery may be advantageous in terms of decreased morbidity and mortality and a lower risk of various complications, such as postoperative arachnoiditis [6] and/or iatrogenic instability, in elderly patients with LSS. It is important to be familiar with the sensitivity and specificity of the available diagnostic tests because a false-positive result may lead to unnecessary surgery and/or expense and additional invasive diagnostic interventions [7].

Barz et al. [8] suggested that the treadmill test has high diagnostic value for identifying LSS as detected on MRI. However, the treadmill test has limited diagnostic ability to determine the level of clinical symptoms in patients with LSS. Therefore, the truly responsible spinal level in patients with NIC should be diagnosed [7,9-15] based on changes in subjective symptoms and objective neurological findings before and after the gait-loading test [9-12,14] and nerve root block [13,14,16-18]. The gait-loading test is a provocation test that is able to demonstrate the spinal level truly responsible for symptoms, which may be masked at rest. Hence, the gait-loading test is indispensable for determination of the involved spinal level in patients with LSS [9-12,14].

The lumbar extension-loading test is also used to evaluate symptoms due to LSS [19]. The lumbar extension-loading test involves maintaining the lumbar region in moderate extension while standing for as long as possible. Changes in subjective symptoms and objective neurological findings can be evaluated after performance of the lumbar extension-loading test. However, the diagnostic value of this test remains uncertain.

The purpose of this study was to compare the diagnostic value of the lumbar extension-loading test with that of the gait-loading test in terms of the ability to determine the responsible spinal level in patients with LSS.

## Methods

This cross-sectional study was approved by the ethics committees of the participating research of institutions of Fukushima Medical University School of Medicine. All patients gave their written informed consents for overall examinations in the hospital, which include both the lumbar extension-loading test and the gait-loading test according to the standard forms. However, all patients never gave a letter of consent for the each loading test, because each test was regular medical examination. The patients were entered into this study prospectively and consecutively.

A total of 116 patients (62 men and 54 women) who were admitted to our hospital with a diagnosis of LSS were included in the study (Table 1). Their mean age was 69.8 years (range, 45–88 years) (Table 1), with most patients being in the 70- to 80-year-old age bracket. Causes of LSS were lumbar spondylosis (n = 76), degenerative spondylolisthesis (n = 37), and degenerative scoliosis (n = 3) (Table 1). An independent radiologist assessed the MRI findings for evidence of LSS, which was characterized by central stenosis and lateral and foraminal stenosis. All patients underwent both the lumbar extension-loading test and the gait-loading test.

**Table 1:** Demographic and clinical characteristics of patients

**Age in years**	**69.8 (45–88)**
**Sex**	
Male	62
Female	54
**Cause of lumbar spinal stenosis**	
Lumbar spondylitis	76
Degenerative spondylolisthesis	37
Degenerative scoliosis	3

Age is given as mean (range). All other data are given as number of patients.

The inclusion criteria for all patients were 1) intermittent claudication, 2) cognitive capability to complete all required inquires, and 3) a diagnosis of LSS characterized by central stenosis and lateral and foraminal stenosis on MRI (Table 2). The exclusion criteria for all patients were 1) predominantly axial spinal pain, 2) known peripheral neuropathy, 3) an ankle brachial pressure index of <0.9, 4) a history of worker’s compensation or disability issues, and 5) combined cervical and/or thoracic myelopathy (Table 2).

**Table 2:** Inclusion and exclusion criteria

**Inclusion criteria**	
1)	Intermittent claudication
2)	Cognitive capability to complete all inquires
3)	Diagnosis of lumbar spinal stenosis characterized by central stenosis and lateral and foraminal stenosis on magnetic resonance imaging
**Exclusion criteria**	
1)	Predominantly axial spinal pain
2)	Known peripheral neuropathy
3)	Ankle brachial pressure index of <0.9
4)	History of worker’s compensation or disability issues
5)	Combined cervical and/or thoracic myelopathy

### Lumbar extension-loading test

The examiner asked the patient to maintain slightly more than moderate lumbar extension (angle of 10°–30°) until their symptoms worsened or they felt tired. Patients were asked to describe new subjective symptoms when they occurred until the test was stopped. The examiner recorded the patient’s subjective symptoms and objective neurological findings immediately after stopping the test while the standing posture was maintained.

### Gait-loading test

The examiner asked the patient to continue walking as long as possible at a constant speed around a flat 100-m circuit in the ward. While walking, the patient was asked to maintain a neutral or slightly extended lumbar position. Patients were asked to describe new subjective symptoms when they occurred until the test was stopped. The test was stopped when patients reported worsening of subjective symptoms. The examiner recorded the subjective symptoms and objective neurological findings immediately after stopping the test while the standing posture was maintained [11,20]. The time and distance walked were also recorded; the walking distance was calculated as 100 m (lap distance) × the number of laps.

### Evaluation of subjective symptoms and objective neurological findings

The orthopedic surgeons administered the two loading tests and evaluated the subjective symptoms and objective neurological findings. Although some orthopedic surgeons were involved in both loading tests, the two loading tests of a given patient were examined by one orthopedic surgeon. The two loading tests were conducted on separate days with a 1-day interval between each test. All patients underwent the gait-loading test first and the lumbar extension-loading test second. Changes in subjective symptoms and objective neurological findings after the two tests were analyzed. The degrees and ranges of subjective symptoms were graded using a visual analog scale. First, data were analyzed with the aim of evaluating the ability of the lumbar extension-loading test to determine the spinal level responsible for LSS. Second, the lumbar extension-loading test was compared with the gait-loading test. Motor function was categorized from 0 to 5 with manual muscle testing. Muscle power was also graded using manual muscle testing. Sensory function was evaluated based on the degree and range of hypalgesia with the pinprick method. The patellar tendon reflex and the Achilles tendon reflex were assessed as normal, or absent.

The patients were classified into three groups (Groups A, B, and C) according to their changes in subjective symptoms and objective neurological findings following the two tests. In Group A, new subjective symptoms and/or objective neurological findings that were not present at rest occurred after lumbar-extension and/or gait-loading. In Group B, the severity of subjective symptoms and/or objective neurological findings increased after the lumbar-extension and/or gait-loading test, but these symptoms and/or findings were not worse than those before the loading tests. Group C showed no changes in subjective symptoms or objective neurological findings after both loading tests.

### Determination of the spinal level responsible for LSS

Hypalgesia of the medial knee [16,21], motor weakness of the quadriceps muscle, and a reduced or absent patellar tendon reflex were considered to indicate a second, third, or fourth lumbar (L2–4) radiculopathy. Hypalgesia of the area from the lateral lower leg to the medial dorsal foot [16,21] and motor weakness of the tibialis anterior, extensor hallucis longus, and extensor digitorum longus muscles were assessed as a fifth lumbar (L5) radiculopathy. Finally, hypalgesia of the lateral foot and sole [16,21]; motor weakness of the gastrocnemius, flexor hallucis longus, and flexor digitorum longus muscles; and a reduced or absent Achilles tendon reflex were assessed as a first sacral (S1) radiculopathy.

### Comparison of subjective symptoms and objective neurological findings between the lumbar extension-loading test and the gait-loading test

The consistency rate (percentage) of each loading test was calculated after evaluation of the changes in subjective symptoms and objective neurological findings. Statistical analyses of the consistency rate (percentage) of each loading test after evaluation of the changes in subjective symptoms and objective neurological findings were performed with Fisher’s exact test. The results were considered to be significant if the obtained p values were <0.05. All statistical analyses were performed using the StatView 5.0 statistical software package (SAS Inc., Cary, NC, USA).

## Results

The lumbar extension posture was maintained for a minimum of 1.0 minute and a maximum of 27.0 minutes (mean, 5.6 minutes) during the lumbar extension-loading test. The walking time ranged from 0.5 to 35.0 minutes (mean, 6.5 minutes) and the walking distance ranged from 100.0 to 1000.0 m (mean, 141.3 m) during the gait-loading test.

### Changes in subjective symptoms

After the lumbar extension-loading test, 46 patients (40%) were assigned to Group A, 66 (57%) to Group B, and 4 (3%) to Group C (Table 3). One patient was allocated to Group C in the gait-loading test, but to Group A in the lumbar extension-loading test. All other patients remained in the same categories after each test. Changes in symptoms were 99% consistent between the two tests. There was no significant difference between the two tests (p = 0.9261).

**Table 3:** Analysis of subjective symptoms and objective neurological findings in the four groups

		**Group A**	**Group B**	**Group C**
Subjective symptoms	E-L	46 (40)	66 (57)	4 (3)
	G-L	47 (40)	66 (57)	3 (3)
Motor	E-L	29 (25)	49 (42)	38 (33)
	G-L	29 (25)	51 (44)	36 (31)
Sensory	E-L	5 (5)	37 (31)	74 (64)
	G-L	5 (5)	37 (31)	74 (64)
Reflex	E-L	32 (28)	-	84 (72)
	G-L	31 (27)	-	85 (73)

Data are given as n (%).

E-L: lumbar extension-loading test, G-L: gait-loading test.

Group A: New subjective symptoms and/or objective neurological findings that were not present at rest occurred after lumbar-extension and/or gait-loading.

Group B: The severity of subjective symptoms and/or objective neurological findings increased after the lumbar-extension and/or gait-loading test, but these symptoms and/or findings did not worsen.

Group C: No changes in subjective symptoms or objective neurological findings after both loading tests.

### Changes in objective neurological findings

Motor: After the lumbar extension-loading test, 29 patients (25%) were assigned to Group A, 49 (42%) to Group B, and 38 (33%) to Group C (Table 3). Two patients were allocated to Group B in the gait-loading test, but to Group C in the lumbar extension-loading test. Changes in motor findings showed 98% consistency between the two tests. There was no significant difference between the two tests (p = 0.9541).

Sensory: After both tests, 5 patients (5%) were assigned to Group A, 37 (31%) to Group B, and 74 (64%) to Group C (Table 3). Changes in hypalgesia showed 100% consistency between the two tests.

Reflexes: After the lumbar extension-loading test, 32 patients (28%) were assigned to Group A and 84 (72%) to Group C (Table 3). After the gait-loading test, 31 patients (27%) were allocated to Group A and 85 (73%) to Group C (Table 3). One patient was assigned to Group C in the gait-loading test, but to Group A in the lumbar extension-loading test. Changes in reflexes were 99% consistent between the two tests. There was no significant difference between the two tests (p = 0.8826).

### Changes in spinal level responsible for LSS

Of the 46 patients in the lumbar extension-loading test and 47 patients in the gait-loading test in Group A, both loading tests identified the newly involved responsible spinal level in 24 patients who did not have subjective symptoms at rest. Of these 24 patients, 12 had suspected L4–5 spinal level involvement before the lumbar extension-loading test, but were determined to have L3–4 and L4–5 spinal level involvement or focal L4 level involvement after the lumbar extension-loading test (Table 4). This new L4 radiculopathy presented as follows: 11 of these patients had patellar tendon reflex changes, 7 showed alterations in the extent of hypalgesia of the medial knee, 9 had changes in motor weakness at the quadriceps muscle, and all 12 had alterations in symptoms. None of these 12 patients showed differences in the evaluation results between before and after the gait-loading test. In the remaining 12 of the 24 above-described patients, the L3–4 and L4–5 levels were suspected to be responsible based on the evaluation before the lumbar extension-loading test (Table 4). However, the spinal level truly responsible for symptoms was likely to be the L3–4, L4–5, and more cranial levels based on the post-test evaluation of the extent of hypalgesia in the area from the medial knee to the entire anterior thigh, motor weakness of the quadriceps and iliopsoas muscles, and corresponding anatomical neural compression observed with MRI and/or myelography (Table 4). In the final diagnosis, 2 of the 12 patients were determined to have L1–2, 2–3, 3–4, and 4–5 levels of involvement, and 10 of the 12 patients were determined to have L2–3, 3–4, and 4–5 levels of involvement. None of these 12 patients showed differences in the evaluation results between before and after the gait-loading test.

**Table 4:** Changes in spinal levels responsible for lumbar spinal stenosis in the two tests

	**E-L**		**G-L**	
	**12 patients**	**12 patients**	**12 patients**	**12 patients**
Pretest evaluation	L4–5	L3–4, L4–5	L4–5	L3–4, L4–5
Post-test evaluation	L3–4, L4–5	L3–4, 4–5, and more cranial levels	L3–4, L4–5	L3–4, 4–5, and more cranial levels

E-L: lumbar extension-loading test, G-L: gait-loading test.

## Discussion

The present study demonstrated the following findings regarding consistency between the two tests: a) changes in subjective symptoms were in 99% agreement (p = 0.9261), b) changes in muscle power were in 98% agreement (p = 0.9541), c) changes in hypalgesia were in 100% agreement, d) changes in reflexes were in 99% agreement (p = 0.8826), and e) changes in the spinal level responsible for LSS were 100% consistent between the two tests. These findings suggest that as a provocation test, the lumbar extension-loading test may be comparable with the gait-loading test.

For correct diagnosis of the responsible spinal level, it may be necessary to accurately analyze not only LSS on images, but also the patient’s subjective symptoms, physical findings, and objective neurological findings caused by LSS. The lumbar extension-loading test may also have a comparable role in understanding the pathology of LSS and in determining the spinal level truly responsible for symptoms masked at rest. Therefore, the lumbar extension-loading test may be substituted for the gait-loading test and therefore have diagnostic value for patients with LSS.

**Figure 1:**
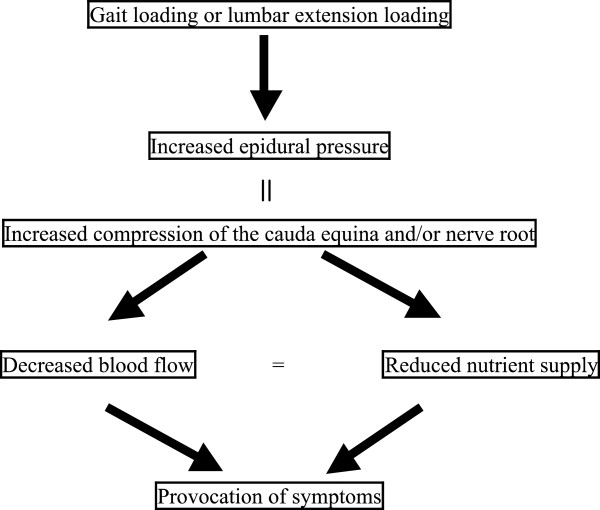
**Mechanisms underlying the gait-loading and lumbar extension-loading tests.** Subjective symptoms after walking and those triggered by lumbar extension are believed to be similarly provoked by the comparative lack of nutrient supply to the cauda equina after the blood flow to this structure decreases. This may be related to venous congestion leading to increased pressure in the epidural space.

It may be considered that NIC is provoked by an alteration of the microcirculation supplying nerves, causing a subsequent lack of nutrient supply that can result from persistent nerve root and/or cauda equina compression during walking or the postural change involved in lumbar extension [21-24]. The development of NIC may be related to venous congestion caused by increased pressure in the epidural space [25] (Figure 1). The gait-loading test [11,12] has been applied to the evaluation of NIC [26]. It is considered to be an appropriate method with which to investigate the pathophysiology of NIC because the subjective symptoms of NIC are provoked during walking. The gait-loading test suggests the truly involved spinal level when changes in the subjective symptoms and objective neurological findings after the gait-loading test are analyzed [11,12].

However, the gait-loading test is time-consuming and requires space for walking. A more efficient method for evaluation of NIC is therefore needed. The lumbar extension-loading test is a promising candidate because it requires less space and time to perform. Katz reported that changes in subjective symptoms after maintaining a lumbar extension posture are useful for diagnosis of the involved spinal level [15]. However, there has been no published report on the diagnosis of the truly involved spinal level based on both subjective and objective neurological findings examined before and after a provocation test.

Intraspinal pressure (epidural pressure) might play an important role in the mechanism of development of symptoms provoked by the gait-loading and lumbar extension-loading tests [25,27-29]. Clinically, a different mechanism of cauda equina compression may exist between walking and lumbar extension. Walking may mimic the state of intermittent cauda equina compression, while lumbar extension may mimic the state of continuous compression. A previous experimental study compared the reduction in the muscle action potential amplitude between intermittent and continuous cauda equina compression using a porcine model [30]. The study suggested that the muscle action potential amplitude reduction was proportional to the applied pressure [30]. That is to say, the functional impairment was more pronounced when compression was applied with one site of intermittent compression and one site of continuous compression than with two sites with intermittent compression.

The lumbar extension-loading test has several limitations. First, it has never been used alone to conclusively determine the spinal level responsible for LSS. Although subjective symptoms and objective neurological findings provoked by the lumbar extension-loading test are useful for estimating the responsible level, a nerve root block may be necessary for a conclusive diagnosis of the responsible spinal level. Second, the test has never been validated. It is difficult to evaluate the reliability of the lumbar extension-loading test by means of retesting because this would be too cumbersome and painful for patients with LSS. Moreover, it is difficult to evaluate the validity of the lumbar extension-loading test because there is no universally accepted, definitive, objective diagnostic criterion for determination of the truly responsible level. Third, observation bias is possible, particularly if observers differ in terms of their explanations to patients of how to perform the test, how they ask about symptoms, and how they examine the patient. Such observation bias might be reduced if the clinician responsible for performing the test ensures uniformity in the test procedure, explanation and instructions provided to patients, examination method, and methods of inquiring about the patient’s subjective symptoms and determining their objective neurological findings. However, complete prevention of such observation bias appears to be difficult. Fourth, different results of the manual muscle test were readily obtained because the two examiners evaluated each loading test. Fifth, the gait-loading test was performed before the lumbar extension-loading test. This may have introduced some bias in terms of the responsible spinal level because of the preloading test results. Finally, we did not unify the examination time from morning to night for each test. This may have contributed to the different results of the analysis of the responsible spinal level.

## Conclusions

The lumbar extension-loading test and the gait-loading test were compared in terms of efficacy of diagnosis of the spinal level responsible for LSS. Both tests were evaluated in terms of changes in subjective symptoms, changes in objective neurological findings, and changes in the spinal level considered to be responsible. The lumbar extension-loading test is useful for understanding the pathology of LSS and determining the truly responsible spinal level when symptoms are masked at rest. Surgery at only the involved spinal level may prevent unnecessary complications during and after surgery; moreover, appropriate decompression may prevent failed back syndrome. Performance of the lumbar extension-loading test and the gait-loading test in cooperation with a therapist is recommended to diagnose the truly responsible level in patients with LSS.

## Abbreviations

NIC: Neurogenic intermittent claudication; LSS: Lumbar spinal stenosis; MRI: Magnetic resonance imaging; L5: A fifth lumbar; S1: A first sacral; E-L: Lumbar extension-loading test; G-L: Gait-loading test.

## Competing interests

The authors declare that they have no competing interests.

## Authors' contributions

N T, S Ki, S Y, K O, S Ko. We all have substantial contributions to the conception and design of the work, one author (NT) acquired the data, four authors (NT, SY, KO, and SKo) analyzed the data, all authors contributed to the interpretation of data for the work, three authors (SKo, Ski, and SY) drafted the manuscript and all authors critically appraised the content of the manuscript. All authors read and approved the final manuscript.

## Pre-publication history

The pre-publication history for this paper can be accessed here:

http://www.biomedcentral.com/1471-2474/15/259/prepub
